# Anti-Inflammatory Effects of Extracellular Vesicles from *Ecklonia cava* on 12-O-Tetradecanoylphorbol-13-Acetate-Induced Skin Inflammation in Mice

**DOI:** 10.3390/ijms252312522

**Published:** 2024-11-21

**Authors:** Geebum Kim, So Young Lee, Seyeon Oh, Jong-Won Jang, Jehyuk Lee, Hyun-Seok Kim, Kuk Hui Son, Kyunghee Byun

**Affiliations:** 1 Misogain Dermatology Clinic, Gimpo 10108, Republic of Korea; drkgb77@gmail.com; 2 Department of Thoracic and Cardiovascular Surgery, Gachon University Gil Medical Center, Gachon University, Incheon 21565, Republic of Korea; faustina117@gilhospital.com; 3 Functional Cellular Networks Laboratory, Lee Gil Ya Cancer and Diabetes Institute, Gachon University, Incheon 21999, Republic of Korea; seyeon8965@gmail.com (S.O.); jh58333@gachon.ac.kr (J.-W.J.); 4 Department of Health Sciences and Technology, Gachon Advanced Institute for Health & Sciences and Technology (GAIHST), Gachon University, Incheon 21999, Republic of Korea; 5 Department of Anatomy & Cell Biology, College of Medicine, Gachon University, Incheon 21936, Republic of Korea; doctorbom@gachon.ac.kr; 6 Doctorbom Clinic, Seoul 06614, Republic of Korea; 7 Kim Hyun Seok Plastic Surgery Clinic, Seoul 06030, Republic of Korea; khsps7799@gmail.com

**Keywords:** *Ecklonia cava* extract, chronic skin inflammation, NLRP3 inflammasome

## Abstract

Steroids, which are often used to treat the inflammation associated with various skin diseases, have several negative side effects. As *Ecklonia cava* extract has anti-inflammatory effects in various diseases, we evaluated the efficacy of *Ecklonia cava*-derived extracellular vesicles (EVEs) in decreasing 12-O-tetradecanoylphorbol-13-acetate (TPA)-induced inflammation. We determined the effect of the EVEs on the TLR4/NF-κB/NLRP3 inflammasome in human keratinocytes and mouse ear skin. TPA-treated human keratinocytes showed an increased expression of TLR4 and its ligands HMGB1 and S100A8. TPA also increased the expression of (1) NF-κB; (2) the NLRP3 inflammasome components NLRP3, ASC, and caspase 1; and (3) the pyroptosis-related factors GSDMD-NT, IL-18, and IL-1β. However, the expression of these molecules decreased in the TPA-treated human keratinocytes after EVE treatment. Similar to the in vitro results, TPA increased the expression of these molecules in mouse ear skin, and EVE treatment decreased their expression. The TPA treatment of skin increased edema, redness, neutrophil infiltration, and epidermal thickness, and EVE reduced these symptoms of inflammation. In conclusion, the EVEs decreased TPA-induced skin inflammation, which was associated with a decrease in the TLR4/NF-κB/NLRP3 inflammasome.

## 1. Introduction

Inflammation is a defense mechanism against stimuli such as the invasion of pathogens [1]. Acute inflammation during the wound healing process is needed for the removal of pathogens [2]; however, uncontrolled or chronic inflammation leads to tissue injuries and even necrosis [3]. Chronic inflammation is a characteristic of various skin maladies, such as dermatitis and acne [4]. Steroids attenuate the edema, pain, and heat of inflammation; however, the long-term use of steroids causes various complications, including skin atrophy and an increased risk of opportunistic infections [5].

In chronic inflammation, various immune cells, such as macrophages and lymphocytes, which have Toll-like receptor (TLR)4 on their plasma membranes, upregulate various inflammatory factors, such as interleukin (IL)-6, tumor necrosis factor-alpha (TNF)-α, and prostaglandin E2 [6].

The topical application of 12-O-tetradecanoylphorbol-13-acetate (TPA), which induces skin inflammation in animal models [7], increases TLR4, TNF, and various inflammatory cytokines [8,9,10,11]. TPA also increases high mobility group box-1 protein (HMGB1), which is a TLR4 ligand in keratinocytes, and it promotes inflammation pathways such as nuclear factor-κB (NF-κB), which is downstream of TLR4 [9,12].

The damage-associated molecular pattern (DAMP) molecules S100A8 and S100A9, which also activate TLR4 and NF-κB [13,14], increase in inflammatory skin diseases such as psoriasis and atopic dermatitis [15,16,17]. DAMPs promote the formation of the NOD-like receptor protein 3 (NLRP3) inflammasome through TLR4 and NF-κB [18].

The inflammasome is an intracellular protein complex that activates caspase-1 and the inflammatory response [19]. Inflammasomes have sensor proteins, germline-encoded pattern-recognition receptors (PRRs), which can recognize DAMPs [20]. NLRP3 is one of the PRRs that form the NLRP3 inflammasome [21]. The NLRP3 inflammasome is essential for the host’s immune defense against viruses or bacteria; however, it also participates in the development of diseases such as cardiovascular disease and diabetes by increasing chronic inflammation [22,23].

The NLRP3 inflammasome complex comprises NLRP3 (a sensor), the apoptosis-associated speck-like protein (ASC) (an adaptor), and caspase 1 (an effector) [24,25]. In the NLRP3 inflammasome, pro-caspase 1 is converted to active caspase 1 via autoproteolysis [26,27]. Activated caspase 1 cleaves pro-IL-1β and pro-IL-18 into the active forms IL-1β and IL-18 [26,28].

Pyroptosis is a programmed cell death mechanism, and it is mediated by gasdermin [28]. Pyroptosis has some similarities to apoptosis, which is also a programmed cell death mechanism, as DNA damage and chromatin condensation are observed during both pyroptosis and apoptosis. However, pyroptosis induces cell swelling and rupture, which induces inflammation differently from apoptosis [29,30,31,32].

Activated caspase 1 also cleaves membrane pore-forming gasdermin D (GSDMD), releasing the GSDMD-N-terminal domain (GSDMD-NT), which generates cell membrane pores [26,28]. The pores allow water to enter the cells, resulting in cell swelling and osmotic lysis, which induces pyroptosis [33,34]. Pyroptosis leads to the release of intracellular molecules such as IL-1β and IL-18, which aggravate inflammation [35,36].

The NLRP3 inflammasome is involved in the pathophysiology of various skin diseases, such as psoriasis, urticaria, and bullous pemphigoid [37]. Pyroptosis is also related to various skin diseases such as psoriasis, vitiligo, and atopic dermatitis [38]. Recently, extracellular vesicles (EVs) have been evaluated as immunomodulators that could decrease skin inflammatory diseases [39]. EVs, which have a 30–2000 nm diameter, are phospholipid bilayer membrane-bound particles derived from cell membranes that are released into the extracellular space by most cells [28]. EVs contain RNAs, lipids, and proteins that are involved in the immune response, cell survival, and cell proliferation [40,41,42,43,44,45]. EVs from immune cells affect skin diseases such as psoriasis and atopic dermatitis [39], and the use of EVs from various cells has been studied as a treatment for inflammatory diseases. Plant-derived EVs may mitigate inflammation; for example, ginger-derived EVs decrease NLRP1 inflammasome activation in colitis [46,47,48]. Ginger-derived EVs also decrease TLR4 activation in lung inflammation induced by severe acute respiratory syndrome coronavirus-2 [49]. Shiitake mushroom-derived EVs decrease NLRP3 inflammasome-induced inflammation in acute liver injury [50,51].

Extracts of *Ecklonia cava*, a brown alga that grows mainly in Korea and Japan [52], have anti-inflammatory effects [53]. Previously, we reported that *E. cava*-derived EVs (EVEs) decrease the expression of TNF-α, mitogen activated protein kinase (MAPK), and NF-κB, which decrease oxidative stress in aged animal skin, leading to increased collagen fiber accumulation [54]. Moreover, EVEs decrease melanogenesis by decreasing the TXNIP/NLRP3/IL-18 pathway in ultraviolet-irradiated animal skin [55]. Although EVEs decrease oxidative stress, NF-κB, and the NLRP3 inflammasome in animal skin, it is not known whether they decrease TPA-induced skin inflammation. In this study, we found that EVEs decreased skin inflammation by reducing HMGB1 and S100A8 and decreasing TLR4 expression, which then inhibited pyroptosis. We hypothesized that EVEs inhibit the TLR4/NF-κB/NLRP3 inflammasome, which decreases IL-1β, IL-18, and pyroptosis, thereby attenuating TPA-induced skin inflammation.

## 2. Results

### 2.1. EVEs Decreased the Expression of HMGB1, S100A8, TLR4, and NF-κB in TPA-Treated Human Keratinocytes

The EVE treatment concentration was determined by its effect on cell viability and its ability to inhibit HMGB1 and S100A8 expression. EVEs were administered as a solution, which was mixed with distilled water (DW). A cryo-transmission electron microscopy (cryo-TEM) image showed the EVEs as round particles with a double-layered membrane in the DW mixture (Figure S1). Human keratinocyte viability after EVE treatments of 1–4 mg/mL was not significantly different from that after treatment with phosphate-buffered saline (PBS)-treated control cells. However, human keratinocyte viability decreased with 5 mg/mL of the EVEs (Figure S2). The expression of HMGB1 and S100A8 increased in TPA-treated human keratinocytes, and EVE concentrations of 0.025, 0.05, 0.1, and 0.2 mg/mL decreased their expression; however, the effect was similar for EVE concentrations higher than 0.05 mg/mL (Figure S3). Thus, we chose an EVE concentration of 0.05 mg/mL for subsequent in vitro experiments.

We compared EVE treatment with corticosteroid dexamethasone (DXA) in TPA-treated human keratinocytes (Figure S4). The treatment concentration of DXA was determined by previous studies [56,57].

**Figure 1:**
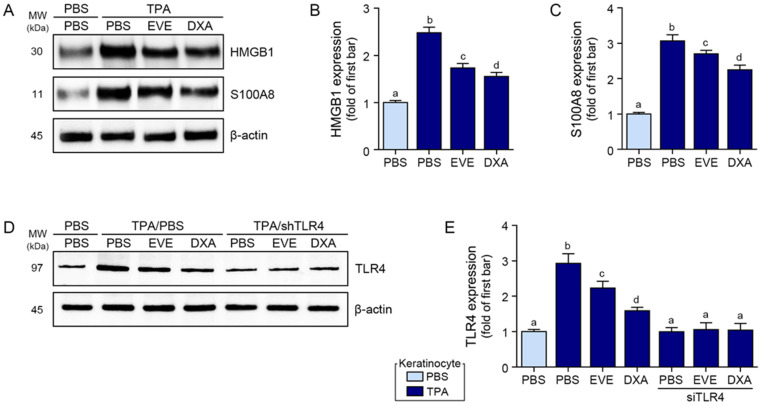
Regulation of HMGB1, S100A8, and TLR4 by EVEs in TPA-treated human keratinocytes. (**A**) Western blot detection of HMGB1 and S100A8 expressions in TPA-treated human keratinocytes subjected to EVEs or DXA. (**B**,**C**) Quantification analysis of (**B**) HMGB1 and (**C**) S100A8 with (**A**) Western blot images using Image J software version 1.53. (**D**,**E**) TLR4 protein expression in TPA-treated human keratinocytes subjected to EVEs or DXA after TLR4 silencing. β-actin was determined as loading control. Human keratinocytes were treated with 100 nM TPA for 4 h, followed by 48 h incubation with PBS, EVEs (0.05 mg/mL), or DXA (0.001 mM). TLR4 knockdown was achieved by transfecting 500 ng of TLR4 shRNA plasmid for 24 h prior to treatment. Data are presented as mean ± SD of three independent experiments. *p* < 0.05; a–d; same letters indicate nonsignificant differences between groups, as determined by multiple comparisons (Mann–Whitney U test). DXA, dexamethasone; EVE, extracellular vesicle from *E. cava*; HMGB1, high mobility-group box-1 protein; ICC, immunocytochemistry; PBS, phosphate-buffered saline; SD, standard deviation; TLR4, Toll-like receptor 4; TPA, 12-O-tetradecanoylphorbol-13-acetate.

**Figure 2:**
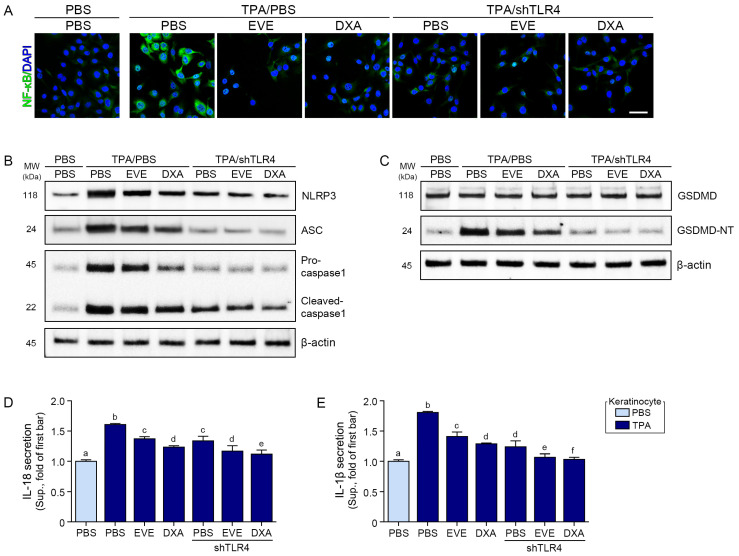
Regulation of NF-κB, NLRP3 inflammasome, and pyroptosis by EVEs in TPA-treated human keratinocytes. (**A**) Representative images of NF-κB (green) immunofluorescence staining in TPA-treated human keratinocytes subjected to EVEs or DXA after TLR4 silencing. Nuclei were stained with DAPI (blue). Scale bar = 50 μm. (**B**) Western blot detection of NLRP3, ASC, pro-caspase 1, and cleaved-caspase expressions in TPA-treated human keratinocytes subjected to EVEs or DXA after TLR4 silencing. (**C**) Western blot detection of GSDMD and GSDMD-NT expressions in TPA-treated human keratinocytes subjected to EVEs or DXA after TLR4 silencing. β-actin was determined as loading control. (**D**,**E**) Quantification analysis of IL-18 and IL-1β secretions in TPA-treated human keratinocytes subjected to EVEs or DXA after TLR4 silencing using ELISA. Human keratinocytes were treated with 100 nM TPA for 4 h, followed by 48 h incubation with PBS, EVEs (0.05 mg/mL), or DXA (0.001 mM). TLR4 knockdown was performed by transfecting 500 ng of TLR4 shRNA plasmid for 24 h before treatment. Data are presented as mean ± SD of three independent experiments. *p* < 0.05; a–f; same letters indicate nonsignificant differences between groups, as determined by multiple comparisons (Mann–Whitney U test). ASC, apoptosis-associated speck-like protein; DXA, dexamethasone; ELISA, enzyme-linked immunosorbent assay; EVE, extracellular vesicle from *E. cava*; GSDMD, gasdermin D; GSDMD-NT, gasdermin D N-terminal domain; IL, interleukin; NLRP3, NOD-like receptor protein 3; SD, standard deviation; TPA, 12-O-tetradecanoylphorbol-13-acetate.

The TPA-induced increase in the expression of HMGB1 and S100A8 was reduced by 0.05 mg/mL of the EVEs and 0.001 mM of DXA; however, the reduction by DXA was significantly better than that by the EVEs (Figure 1A–C). The TPA-induced increase in the expression of TLR4 was reduced by the EVEs and DXA; however, the reduction by DXA was better than that by the EVEs (Figure 1D,E). The activation of NF-κB was evaluated via the translocation of NF-κB. TPA increased the nuclear NF-κB in the human keratinocytes, which was reduced by EVE and DXA treatment; however, the reduction by DXA was significantly better than that by the EVEs (Figure 2A and Figure S5A).

As we hypothesized that EVEs inhibit the TLR4/NF-κB/NLRP3 inflammasome, we evaluated whether they are involved in decreasing TLR4 by comparing the activity of NF-κB between EVE-treated human keratinocytes and TLR4-silenced human keratinocytes. The expression of TLR4 in the TLR4-silenced human keratinocytes was similar to that in the PBS-treated control human keratinocytes (Figure 1D,E). Upon the silencing of TLR4, the TPA-induced increase in NF-κB activity decreased more than in the EVE-treated normal human keratinocytes. When the EVEs and DXA were used to treat the TLR4-silenced human keratinocytes, NF-κB activity decreased more than when PBS was used to treat the TLR4-silenced human keratinocytes (Figure 2A and Figure S5A). These results suggest that the EVEs decreased NF-κB activity by modulating TLR4.

### 2.2. EVEs Decreased the NLRP3 Inflammasome and Pyroptosis in TPA-Treated Human Keratinocytes

TPA treatment increased the expression of the NLRP3 inflammasome components NLRP3, ASC, pro-caspase 1, and cleaved-caspase 1. Although the EVEs and DXA decreased their expression, DXA was more effective (Figure 2B and Figure S5B–E). Pyroptosis, as measured by the expression of cleaved GSDMD (GSDMD-NT), IL-18, and IL-1β, increased after TPA treatment but was reduced by EVEs and DXA; however, DXA was more effective in reducing their expression than the EVEs (Figure 2C–E and Figure S5F).

TLR4 silencing decreased NLRP3, ASC, pro-caspase 1, and cleaved-caspase 1 more than EVE or DXA treatment (Figure 2B and Figure S5B–E). TLR4 silencing also decreased GSDMD-NT, IL-18, and IL-1β more than EVE or DXA treatment (Figure 2C–E and Figure S5F). These results suggest that EVEs and DXA are involved in decreasing NLRP3 inflammasome formation and pyroptosis through their modulation of TLR4.

### 2.3. EVEs Decreased the Expression of HMGB1, S100A8, TLR4, and NF-κB in TPA-Treated Animal Skin

**Figure 3:**
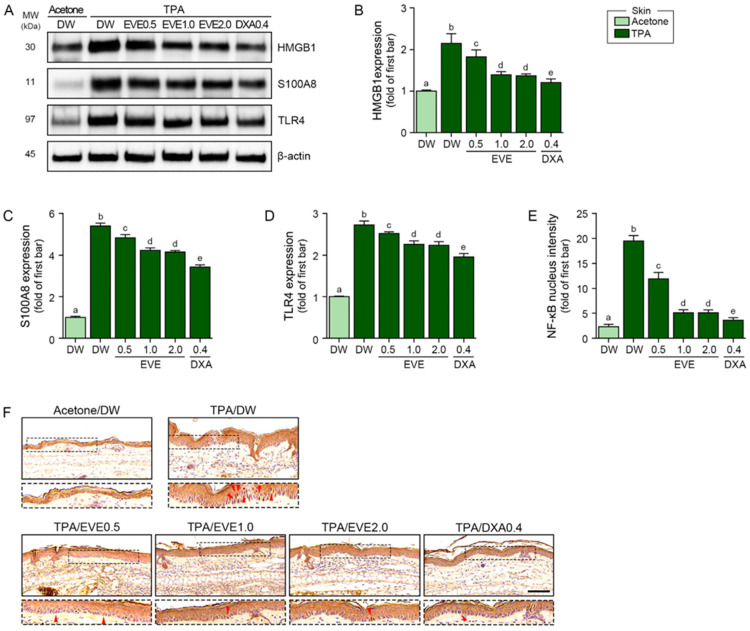
Regulation of HMGB1, S100A8, TLR4, and NF-κB by EVEs in TPA-treated mouse ears. (**A**) Western blot detection of HMGB1, S100A8, and TLR4 expressions in TPA-treated mouse ears subjected to EVEs or DXA. β-actin was determined as loading control. (**B**–**E**) Quantification analysis of (**B**) HMGB1, (**C**) S100A8, and (**D**) TLR4 with (**A**) Western blot images using Image J software. (**F**) Representative images of NF-κB immunohistochemistry staining in TPA-treated mouse ears subjected to EVEs or DXA. Nuclei were stained with hematoxylin (blue). Red arrows indicate positive signals. Scale bar = 100 μm. For each mouse, 50 μM TPA was applied topically to one ear five times at 3-day intervals over 15 days, followed by weekly application of EVEs (0.5, 1.0, or 2.0 mg/mL) or DXA (0.4 mg/kg). Data are presented as mean ± SD of three independent experiments. *p* < 0.05; a–e; same letters indicate nonsignificant differences between groups, as determined by multiple comparisons (Mann–Whitney U test). DW, distilled water; DXA, dexamethasone; EVE, extracellular vesicle from *E. cava*; HMGB1, high mobility group box-1 protein; IHC, immunohistochemistry; NF-κB, nuclear factor-κB; SD, standard deviation; TLR4, Toll-like receptor 4; TPA, 12-O-tetradecanoylphorbol-13-acetate.

To determine whether EVEs decreased the expression of the TLR4 ligands of HMGB1 and S100A8 in a mouse model of TPA-induced skin inflammation, we applied topical TPA to the right ear five times at 3-day intervals and measured the effects of the EVEs and DXA. The TPA-induced increase in the expression of HMGB1 and S100A8 was reduced by the EVEs at 0.5 mg/mL, 1 mg/mL, and 2 mg/mL and by DXA at 0.4 mg/kg, but DXA was more effective (Figure 3A–C). The TPA-induced increase in TLR4 expression was also reduced by the EVEs and DXA, but DXA was more effective (Figure 3A,D). The activation of NF-κB, as measured via the translocation of NF-κB, increased after TPA treatment but was reduced by the EVEs and DXA. However, DXA was more effective than the EVEs in reducing the translocation of NF-κB (Figure 3E,F).

### 2.4. EVEs Decreased the NLRP3 Inflammasome and Pyroptosis in TPA-Treated Animal Skin

**Figure 4:**
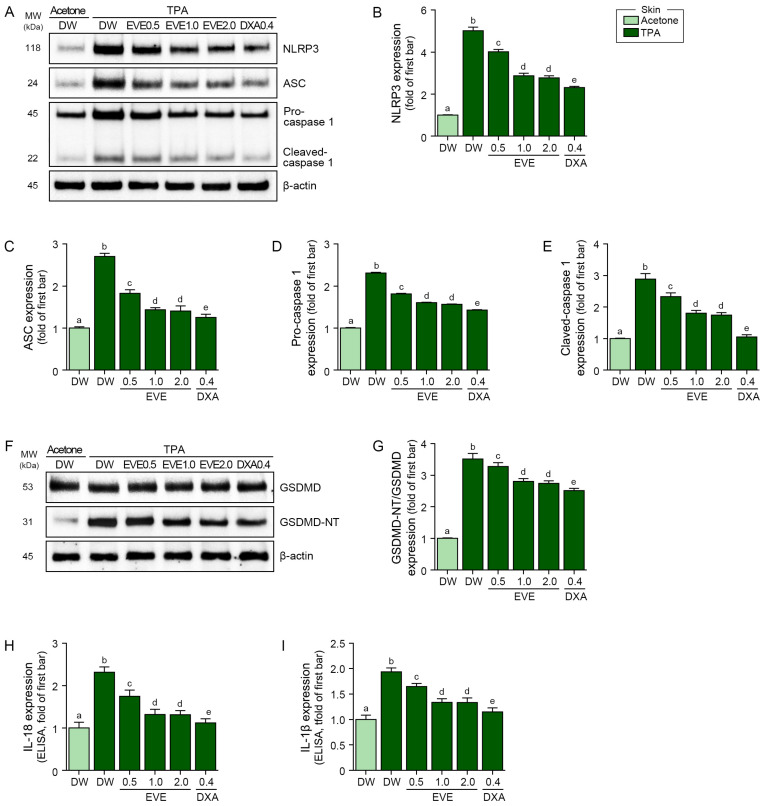
Regulation of NLRP3 inflammasome and pyroptosis by EVEs in TPA-treated mouse ears. (**A**) Western blot detection of NLRP3, ASC, pro-caspase 1, and cleaved-caspase expressions in TPA-treated mouse ears subjected to EVEs or DXA. (**B**–**E**) Quantification analysis of (**B**) NLRP3, (**C**) ASC, (**D**) pro-caspase 1, and (**E**) cleaved-caspase 1 with (**A**) Western blot images using Image J software. (**F**) Western blot detection of GSDMD and GSDMD-NT expressions in TPA-treated mouse ears subjected to EVEs or DXA. β-actin was determined as loading control. (**G**) Quantification analysis of GSDMD-NT with (**F**) Western blot images using Image J software. (**H**,**I**) Quantification analysis of IL-18 and IL-1β in TPA-treated mouse ears subjected to EVEs or DXA using ELISA. For each mouse, 50 μM TPA was applied topically to one ear five times at 3-day intervals over 15 days, with weekly applications of EVEs (0.5, 1.0, or 2.0 mg/mL) or DXA (0.4 mg/kg). Data are presented as mean ± SD of three independent experiments. *p* < 0.05; a–e; same letters indicate nonsignificant differences between groups, as determined by multiple comparisons (Mann–Whitney U test). ASC, apoptosis-associated speck-like protein; DW, distilled water; DXA, dexamethasone; ELISA, enzyme-linked immunosorbent assay; EVE, extracellular vesicle from *E. cava*; GSDMD, gasdermin D; GSDMD-NT, gasdermin D N-terminal domain; IL, interleukin; NLRP3, NOD-like receptor protein 3; SD, standard deviation; TPA, 12-O-tetradecanoylphorbol-13-acetate.

TPA treatment increased the expression of the NLRP3 inflammasome components NLRP3, ASC, pro-caspase 1, and cleaved-caspase 1, and this expression was reduced by the EVEs at 0.5 mg/mL, 1 mg/mL, and 2 mg/mL and by DXA at 0.4 mg/kg. However, DXA was more effective than the EVEs in reducing the NLRP3 inflammasome components (Figure 4A–E). Similarly, TPA treatment increased the expression of GSDMD-NT, IL-18, and IL-1β, which was reduced by the EVEs but was reduced more effectively by DXA (Figure 4F–I).

### 2.5. EVEs Decreased TPA-Induced Inflammation in TPA-Treated Animal Skin

**Figure 5:**
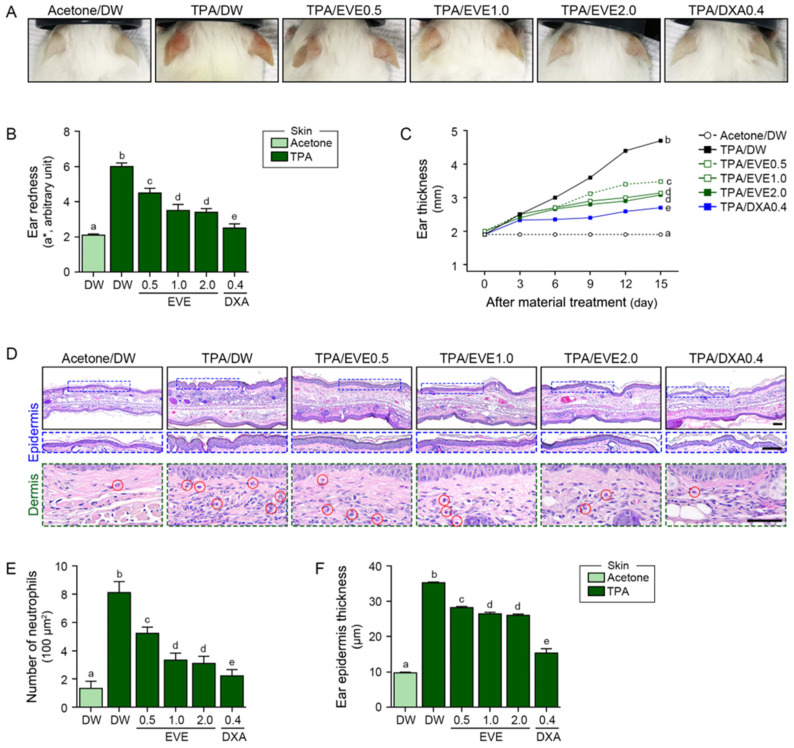
Effect of EVEs on TPA-induced inflammation in TPA-treated mouse ears. (**A**) Representative images of TPA-treated mouse ears subjected to EVEs or DXA at 15 days. (**B**,**C**) (**B**) Ear redness and (**C**) thickness of TPA-treated mouse ears subjected to EVEs or DXA. (**D**) Representative images of hematoxylin and eosin staining in TPA-treated epidermis (blue box) and dermis (green box) of mouse ears subjected to EVEs or DXA. (**E**,**F**) (**E**) Number of neutrophils and (**F**) thickness of epidermis in TPA-treated mouse ears subjected to EVEs or DXA. Red circles indicate positive neutrophil infiltration. TPA (50 μM) was applied topically to each ear five times at 3-day intervals over 15 days. EVEs (0.5, 1.0, or 2.0 mg/mL) or DXA (0.4 mg/kg) were applied weekly. Data are presented as mean ± SD of three independent experiments. *p* < 0.05; a–e; same letters indicate nonsignificant differences between groups, as determined by multiple comparisons (Mann–Whitney U test). DW, distilled water; DXA, dexamethasone; EVE, extracellular vesicle from *E. cava*; SD, standard deviation; TPA, 12-O-tetradecanoylphorbol-13-acetate.

TPA induces skin edema, epidermal hyperplasia, and inflammation [58]. We evaluated skin edema by measuring ear thickness every 3 days, and skin redness was evaluated using a colorimeter. TPA increased both measurements, which decreased after EVE treatment at 0.5 mg/mL, 1 mg/mL, and 2 mg/mL; however, 0.4 mg/kg of DXA [59] provided a greater reduction in edema (Figure 5A–C). Inflammation, measured as neutrophil infiltration using hematoxylin and eosin staining, increased after TPA treatment and was reduced by EVE and DXA treatment; however, DXA treatment was more effective (Figure 5D,E). The overall effects of the EVEs and DXA on epidermal thickness were similar to those on ear thickness, with DXA providing more effective treatment than the EVEs (Figure 5F).

## 3. Discussion

The physical barrier of the skin provides a major defense against external challenges [45]. The outmost layer of the skin is the epidermis, which consists of keratinocytes, melanocytes, and immune cells such as Langerhans cells (LCs) [60]. Harmful agents in the skin lead to acute inflammation, which serves as a defense mechanism [61]. Acute inflammation in the skin increases vascular permeability, which leads to fluid accumulation, and it promotes the infiltration of immune cells, which release inflammatory factors such as cytokines [62,63,64]. These processes induce cardinal inflammatory symptoms, including heat, swelling, redness, and pain [65]. Acute inflammation normally resolves after healing [66]; however, inflammation that persists for months or years is defined as chronic inflammation [67,68]. Chronic inflammatory skin diseases, such as atopic dermatitis, psoriasis, urticaria, lichen planus, and hidradenitis suppurativa, affect 20–25% of the worldwide population [66]. Moreover, skin diseases rank as the fourth leading cause of non-fatal diseases worldwide, indicating a severe disease burden [69]. In 2013, psoriasis alone cost 112 billion USD in the USA [70], and the economic burden of atopic dermatitis was estimated to be 5.297 billion USD in the USA in 2015 [71].

Steroids, which cause skin atrophy, telangiectasia, and stria [72,73], are the primary treatment for chronic inflammation. Depending on steroid potency, patient age, applied surface area, and use duration, topical steroids may lead to systemic complications such as adrenal suppression [73,74]. The discontinuation of topical steroids may result in severe withdrawal symptoms in the skin [75,76]. The prevalence of adverse drug events over 6 months among 1175 topical steroid users in Korea was found to be 7.2% [77]. Additionally, the incidence of adverse drug events was found to be 0.3 cases per 1000 person-days of topical steroid use [77]. The most frequent adverse effects are skin atrophy and hyperpigmentation, which increase with the potency of the steroid and the length of exposure [77]. The steroid use guidelines recommend that high-potency steroids should be used for less than 3–4 weeks [78,79] and low- or moderate-potency steroids for no longer than 3 months [78,79]. Thus, there is a critical need for non-steroidal treatments for chronic skin inflammation.

HMGB1-mediated NF-κB activation is associated with chronic skin inflammation [80]. TLR4, which is expressed in various skin cells such as keratinocytes and fibroblasts, as well as immune cells, increases chronic skin inflammation [65]. TLR4-mediated NF-κB activation leads to the formation of NLRP3 inflammasomes in various inflammatory diseases [81]. In psoriasis, a chronic inflammatory skin disease, IL-1β and IL-18 are increased [82], and excessive NLRP3 and caspase-1 expression levels are associated with its development [83,84].

The use of EVs from various cell sources has been evaluated as a treatment for skin disease. EVs from keratinocytes promote wound healing [45]. Exosomes from adipose-derived stem cells increase wound healing by promoting angiogenesis [85]. EVs from adipose-derived stem cells have been shown to improve skin repair in mouse models of atopic dermatitis [86]. Thus, EVs may serve as an alternative to steroids to treat skin inflammation [87]. Using TEM, we previously reported that the size of EVEs is 30–150 nm [54]. A nanoparticle-tracking analysis showed that the mode of particle size distribution is 81.5 ± 3.1 nm, and the particle concentration is 3.68 × 10^10^ ± 6.40 × 10^9^ particles/mL [54]. EVEs contain nuclear factor erythroid-2-related factor 2 (NRF2) and heat shock protein 70 (HSP70) [55]. HSP70 inhibits MAPK, NADPH oxidases, and NF-κB [88,89]. NRF2 decreases reactive oxygen species, which promotes NLRP3 inflammasomes [90,91]. In fact, EVEs decrease TNF-α, MAPKs, and NF-κB, which are associated with skin rejuvenation in aged animal skin [54]. Because EVEs inhibit various pathways related to inflammation, such as NF-κB [54], it can be inferred that EVEs could decrease chronic skin inflammation. Thus, we hypothesized that EVEs decrease skin inflammation by reducing HMGB1, S100A8, and the TLR4/NF-κB/NLRP3 inflammasome pathway, which eventually decreases TPA-induced skin inflammation. We used an in vitro model of TPA-induced skin inflammation in which TPA was administered to the human keratinocyte.

This study’s results show that HMGB1, S100A8, TLR4, and NF-κB increased in the human keratinocytes treated with TPA. The expression levels of the NLRP3 inflammasome components and pyroptosis-related factors also increased in the TPA-treated human keratinocytes and were reduced by the EVEs. We also silenced TLR4 to evaluate whether EVEs decreased the NF-κB/NLRP3 inflammasome by modulating TLR4. TLR4 silencing decreased the NF-κB/NLRP3 inflammasome pathway, and its decreasing effect was similar to that of the EVE treatment. We also evaluated the EVEs’ effect on skin inflammation in TPA-treated animal skin.

A single topical treatment of 100–200 μL of TPA applied to the ears or dorsal skin of mice leads to acute inflammation 4–8 h later [92,93,94,95], including edema, neutrophil infiltration, erythema, and epidermal cell proliferation [93,94,95,96]. In contrast, the application of 50–200 μM of TPA to ears once or twice a day for 3–10 days [97,98,99,100,101] causes prolonged inflammation and epidermal hyperplasia, characteristic of chronic inflammation [7]. We found that, in the TPA-treated mouse ears, all EVE treatment concentrations decreased HMGB1, S100A8, and TLR4 expression. EVE treatment also decreased the NF-κB/NLRP3 inflammasome. The expression of GSDMD-NT and the mature form of IL-18 and IL-1β, which increased after TPA treatment, decreased after EVE treatment. Ear edema and epidermal hyperplasia also decreased after EVE treatment, but less than after DXA treatment.

Our experimental results suggest that EVEs have the potential to reduce skin inflammation by inhibiting the TLR4/NF-κB/NLRP3 inflammasome pathway. Experimental models that use TCA to cause inflammation in various skin cells or animal skin are widely used, but they have many limitations in mimicking various human skin diseases related to chronic inflammation. Therefore, the notion that EVEs may be clinically effective in human inflammatory skin diseases just because they decrease inflammation in animal skin inflammation models using TCA would be a hasty interpretation. However, as the NLRP3 inflammasome acts as a pathological mechanism in many human skin diseases, our experimental results provide some evidence suggesting that EVEs can be used for the treatment of skin diseases associated with the NLRP3 inflammasome. DXA is the most commonly used potent anti-inflammatory agent, but because it has various complications related to long-term use, efforts to develop drugs that can replace DXA should be continued. In this study, it cannot be argued that EVEs can be used as an alternative to DXA because evaluations of their efficacy and safety, including assessments of their pharmacokinetics and pharmacodynamics, which are essential for developing medicine, were not performed. As DXA can cause serious complications, it is also important to develop products such as cosmetics or drug adjuvants that can reduce the amount of DXA used even if they cannot completely replace DXA. In order to use EVEs as an adjuvant for DXA, evaluations of their long-term toxicity and safety in humans must be performed in the future.

Although mammalian cell-derived EVs could have therapeutic advantages, they are difficult to isolate in sufficient quantities from cells grown in vitro [102]. This results in high costs, although the costs of EV products and their clinical applications depend on the source of the EVs [86]. Thus, plants may provide an economical source of isolated EVs [103]. Edible plants that are abundant, available, biodegradable, and biocompatible are a promising source of EVs [104]. Plant-derived EVs pose little or no risk of transmitting human or zoonotic pathogens and induce a weaker immune reaction than mammalian EVs [105]. Although there is little information on the safety of plant-derived EVs for topical use, the safety of *E. cava* extract has been evaluated. An oral *E. cava* extract produces adverse effects at 2000 mg/kg in Sprague-Dawley rats [106]. A topical *E. cava* extract decreases hair dye-induced oxidative stress without toxicity in zebrafish [107].

However, we did not determine the long-term effects of the EVEs in mice, which should be evaluated. As a high concentration of oral *E. cava* extract is relatively safe for animals [106], the topical application of EVEs might be expected to cause few adverse effects.

We did not evaluate how the EVEs were delivered into the skin in this study. Moreover, we did not evaluate how the EVEs decreased TLR4 expression. There are several possible ways for EVEs to decrease TLR4 expression. As cells can uptake EVs via endocytosis [108], EVEs could be delivered to keratinocytes and decrease TLR4 expression directly in them. In addition, EVs contain various anti-inflammatory factors, and those factors could decrease TLR4 expression. The exact mechanism should be evaluated in future studies.

## 4. Materials and Methods

### 4.1. EVE Preparation

EVEs (EXOBM^TM^) were acquired from SACCI BIO (Seoul, Republic of Korea). The *E. cava* material was extracted at a ratio of 1 part *E. cava* material to 30 parts DW via stirring at 50 °C for 24 h. The extract was centrifuged at 3000× *g* for 30 min at room temperature. The supernatant was transferred to an ultra-clear tube (Beckman, Brea, CA, USA), and vacuoles were removed via high-speed centrifugation at 50,000× *g* for 90 min. The vacuole-free supernatant was centrifuged at 100,000× *g* for 120 min, and the exosomes in the pellet were resuspended in DW and centrifuged again at 100,000× *g* for 120 min to obtain a pellet of clean EVs.

### 4.2. Cryo-Transmission Electron Microscopy (Cryo-TEM)

Cryo-TEM imaging was performed as follows: For cryo-TEM, a Leica EM ACE600 (Leica microsystem, Wetzlar, Germany) was used for the glow discharge of the cryo-TEM grid (200 mesh CF-1.2/1.3 Au, EMS). Then, 5 μL of the EVEs was added onto the grid, and the sample was plunged, frozen, into liquid ethane after the excess fluid was removed via automatic blotting in a Leica EM GP2 (Leica microsystems). The grid was loaded into an Elsa cryo-transfer holder (Gatan, Inc., Pleasanton, CA, USA), and the EVE sample was analyzed with an HT7800 cryo-TEM (HITACHI, Tokyo, Japan) at the Yonsei Biomedical Research Institute, Yonsei University College of Medicine.

### 4.3. In Vitro Experiments

#### 4.3.1. Human Keratinocytes Culture and Cell Viability Assay

HaCaT human keratinocytes were provided by Professor Jeong Hee Hong’s laboratory at Gachon University. The human keratinocytes were cultured in Dulbecco’s modified Eagle’s medium (DMEM; HyClone, Logan, UT, USA) at 37 °C with 5% CO_2_.

To test the cytotoxicity of the EVEs, we seeded the human keratinocytes in a 96-well plate (1 × 10^4^ cells/well). When the cells were 90–100% confluent, they were treated for 24 h with the EVEs at concentrations of 1, 2, 3, 4, and 5 mg/mL (Figure S2A). The medium was removed, and the cells were washed with DPBS (Gibco, Waltham, MA, USA). Then, 10 µL of CCK-8 (Transgenbiotech, Beijing, China) reagent and 90 µL of growth medium were added to each well, and the cells were incubated at 37 °C for 2 h. The optical density at 450 nm was measured using a microplate reader. Each analysis was performed in triplicate. To determine the treatment concentrations of the EVEs, we treated the human keratinocytes with 100 nM TPA for 4 h and then incubated them for 48 h [109] with PBS or with the EVEs at 0.025, 0.05, 0.1, or 0.2 mg/mL, and the cells were lysed for an mRNA analysis (Figure S3A). After finding that 0.5 mg/mL was the treatment concentration of the EVEs, we repeated the TPA treatment of the human keratinocytes and incubated them for 48 h with PBS, 0.05 mg/mL EVEs, or 0.001 mM DXA [56,57]. Control groups without TPA were incubated for 48 h with PBS. After the 48 h of incubation, the cells were collected for a protein or RNA analysis, and the supernatants of the human keratinocytes were also collected (Figure S4).

#### 4.3.2. Transfection of TLR4 shRNA Plasmids into Human Keratinocytes

To investigate the role of TLR4 in the effectiveness of the EVE treatments in TPA-treated human keratinocytes, TLR4 gene expression was suppressed in human keratinocytes (HaCaT). Human keratinocytes at 70–80% confluence were transfected with a TLR4-targeting short hairpin RNA plasmid (TLR4 shRNA plasmid; Santacruz Biotechnology Technology, Dallas, TX, USA) using Lipofectamine 3000 reagent (Invitrogen, Waltham, MA, USA), according to the manufacturer’s protocol. Briefly, 1.5 μL Lipofectamine 3000 reagent, 500 ng TLR4 shRNA plasmid, and 2 μL P3000 reagent were mixed into 100 μL of serum-free medium to generate a DNA–lipid complex. The DNA–lipid complex was incubated at room temperature for 15 min and then diluted in the serum-free medium and cultured with the human keratinocytes for 24 h at 37 °C in an atmosphere containing 5% CO_2_. After 24 h, the cells were treated with TPA, the EVEs, or DXA, as described in Section 4.3.1 (Figure S4).

### 4.4. In Vivo Experiments

#### 4.4.1. Mouse Model and Maintenance

Eight-week-old male ICR mice were obtained from Orient Bio (Sungnam, Republic of Korea) and stabilized in an animal facility for one week before experiments were performed. All animals were housed at 20–24 °C and 45–55% humidity, and they consumed food and water freely. This study was conducted with approval from the Gachon University Animal Experiment Ethics Committee (IACUC, approval number LCDI-2023-0070).

#### 4.4.2. Experimental Design

The stabilized animals were randomly assigned to six groups. Five of these groups were treated with 50 μM TPA applied topically to the ear five times at 3-day intervals, following a previously described method [110]. The EVEs, at a concentration of 0.5, 1.0, or 2.0 mg/mL, or DXA at a dose of 0.4 mg/kg [59], were applied weekly. A total volume of 20 μL of each solution was used per ear for the treatments. After 16 days, the ear skin was collected for analysis.

#### 4.4.3. Ear Redness and Thickness

Ear redness was measured using a CR-10 color reader (Konica Minolta Sensing, Inc., Sakai, Osaka, Japan), with the a* value (indicating redness) assessed in the CIELAB color space (International Commission on Lighting, Vienna, Austria). Measurements were taken five times on day 16, and the average value was calculated.

Ear thickness was measured using a caliper. Measurements were conducted every 3 days over the TPA treatment period, with each measurement repeated five times, and the average value was determined for each animal.

### 4.5. Quantitative Polymerase Chain Reaction (qPCR)

RNA was extracted from cells using an RNAiso kit (TAKARA, Tokyo, Japan), according to the instructions. cDNA synthesis was carried out according to the instructions of a cDNA synthesis kit (TAKARA). For qPCR, 1 µL of cDNA template, 5 µL of SYBR green premix (TAKARA), 0.4 µL each of reverse and forward primers (Table S1), and 3.2 µL of DW were mixed and dispensed in 384 wells (Thermo Fisher Scientific) so that the total volume in each well was 10 µL. The qPCR and a melting curve analysis were performed using a QuantStudio^TM^ 3 real-time PCR instrument (Thermo Fisher Scientific, Waltham, MA, USA), with initial denaturation at 95 °C for 1 min, followed by 40 cycles of denaturation at 95 °C for 5 s, annealing at 60 °C for 30 s, and denaturation at 95 °C for 15 s. A melting curve was produced for a temperature range of 60 °C to 95 °C with an incremental increase of 0.075 °C/s. Gene expression levels were quantified using the comparative cycle threshold (CT) method (ΔΔCT). mRNA levels were normalized to the *ACTB (Human)*/*Actb (mouse)* gene and compared with those in the control.

### 4.6. Western Blot

Proteins were extracted according to the instructions of an EzRIPA buffer kit (ATTO Corporation, Tokyo, Japan). We combined 30 µg of cell lysate or skin protein with a 4× lithium dodecyl sulfate sample buffer (Thermo Fisher Scientific) and 10× sample-reducing agent (Thermo Fisher Scientific). The protein mixture was heated at 70 °C for 10 min, and the denatured proteins were subjected to 10% sodium dodecyl sulfate-polyacrylamide gel electrophoresis (SDS-PAGE) for 25 min at 200 V in MOPS buffer (Invitrogen, Waltham, MA, USA). The separated proteins were transferred to a polyvinylidene fluoride (PVDF) membrane (Millipore, Burlington, MA, USA) using a semi-dry transfer system (ATTO) at a current of 1 A for 10 min. To inhibit non-specific binding, we incubated the PVDF membrane with 5% skim milk (LPS Solution, Daejeon, Republic of Korea) in 0.1% Tween 20 (SPL, Pocheon, Republic of Korea) in Tris-buffered saline (TTBS) at room temperature for 1–2 h. The membrane was washed three times with 0.1% TTBS and incubated with an appropriately diluted primary antibody overnight at 4 °C (Table S2). After three washes with 0.1% TTBS, the membrane was incubated with a horseradish peroxidase-conjugated secondary antibody (1:1000; Vector laboratories, Newark, CA, USA) for 1 h at room temperature. Protein bands were visualized using chemiluminescent solutions and identified with a ChemiDoc Imaging System (Bio-Rad, Hercules, CA, USA). The intensity of the protein bands was quantified using ImageJ software, version 1.53s (NIH). Beta-actin bands were used as an equivalent loading control.

### 4.7. Enzyme-Linked Immunosorbent Assay (ELISA)

Microplates were incubated overnight at 4 °C with 100 nM carbonate (Sigma-Aldrich, St. Louis, MO, USA) and a bicarbonate-mixed buffer (pH 9.6; Sigma-Aldrich) and washed three times with 0.1% Tween 20 in PBS (TPBS) to remove any unattached material. To prevent non-specific protein binding, we incubated the microplates with 5% skim milk (LPS Solution) in 0.1% TPBS overnight at 4 °C. After washing three times with 0.1% TPBS, the keratinocyte supernatant was added to each well and incubated overnight at 4 °C. The wells were washed with 0.1% TPBS and incubated overnight at 4 °C with the primary antibody diluted in PBS (Table S2). After washing with PBS, we added a horseradish peroxidase-conjugated secondary antibody (1:1000; Vector laboratories), and the plate was incubated at room temperature for 4 h. To measure expression, we applied tetramethylbenzidine (TMB) solution (Sigma-Aldrich) to each well, and incubation was carried out for 15–20 min at room temperature. After stopping the reaction with 1 M sulfuric acid (Sigma-Aldrich), we measured the protein using a microplate reader set to 450 nm. Each analysis was performed in triplicate.

### 4.8. Staining

#### 4.8.1. Immunocytochemistry

We seeded 1 × 10^4^ human keratinocytes/well in a confocal cell dish and treated them with TPA for 4 h and with the EVEs or DXA for 48 h. After three washes with PBS, we blocked non-specific binding via incubation with a serum solution for 1 h at room temperature and incubated the slides with a primary antibody overnight at 4 °C (Table S2). The slides were washed with PBS and incubated with the Alexa Fluor^®^ 488 conjugated secondary antibody (Vector laboratories) for 1 h at room temperature. The cells were counterstained with DAPI (Sigma-Aldrich) for 30 s, washed with DW, and mounted in a glycerol mounting solution (Sigma-Aldrich). The stained cells were visualized using an LSM-710 microscope. Each group was compared with the control sample.

#### 4.8.2. Paraffin-Embedded Skin Tissue Block

Skin tissue was fixed in cold 4% paraformaldehyde (Sigma-Aldrich) for 48 h, placed in a cassette, and washed with DW. Using a tissue processor (Leica, Wetzlar, Germany), the sample was soaked sequentially in 95% and 99% ethanol (Duksan, Ansan-si, Republic of Korea), dehydrated, dipped in xylene (Duksan), infiltrated with paraffin (Leica), and made into paraffin blocks in an embedding machine. The blocks were sectioned to a thickness of 7 µm using a microtome (Leica), placed on a coated slide, incubated overnight at 60 °C, and attached to the slide.

#### 4.8.3. Immunohistochemistry

Skin tissue sections were deparaffinized and rehydrated via sequential transfer to xylene and then from 100% to 70% ethanol. The sections were boiled in sodium citrate buffer (pH 6.0; Sigma-Aldrich) in a microwave oven for 5 min and cooled in DW for antigen retrieval. After three PBS washes, we blocked non-specific binding by incubating the sample with serum solution for 1 h at room temperature. The slides were incubated with the primary antibody overnight at 4 °C (Table S2), washed with PBS, and incubated with the biotinylated secondary antibody (Vector laboratories) for 1 h at room temperature. The slides were then rinsed with PBS, incubated with an ABC reagent (Vector laboratories), washed, and incubated with a 3,3′-diaminobenzidine solution (Sigma-Aldrich) for 5 min, resulting in a brown reaction. The slides were counterstained with hematoxylin (KPNT, Cheongju, Republic of Korea) for 30 s, washed with DW, dehydrated, and mounted using a DPX mounting solution (Sigma-Aldrich). Using a slide scanner (Motic Scan Infinity 100; Motic, Beijing, China), we randomly captured images of the stained tissues. Each group was compared with the control sample.

#### 4.8.4. Hematoxylin and Eosin Stain

Hematoxylin and eosin staining was performed according to the manufacturer’s instructions (KPNT). Briefly, skin tissue sections were deparaffinized and rehydrated via sequential transfer to xylene and then from 100% to 70% ethanol. Sections were incubated in hematoxylin solution for 1 min, rinsed three times with DW, incubated in alcoholic eosin solution for 30 s, dehydrated, and then mounted using DPX mounting solution (Sigma-Aldrich). Using a slide scanner (Motic Scan Infinity 100), we randomly captured images of the stained tissues. Each group was compared with the control sample.

### 4.9. Statistical Analysis

The Kruskal–Wallis test was performed to compare the groups, followed by the Mann–Whitney U test for post hoc comparisons. The results are expressed as the mean ± standard deviation. All statistical analyses were performed using SPSS version 26 (IBM, Armonk, NY, USA). Statistical significance is indicated in each figure legend.

## 5. Conclusions

In summary, we investigated the efficacy of EVE treatment in reducing TPA-induced skin inflammation.

The inflammation-reducing effect of EVEs was verified by comprehensive in vitro and in vivo studies, in which they significantly reduced HMGB1 and S100A8, TLR4 ligands that are increased by TPA. The EVEs reduced NF-κB, a downstream pathway of TLR4 and the NLRP3 inflammasome; pyroptosis-related factors, such as GSDMD-NT, IL-1β, and IL-18; and inflammation-related skin changes, such as redness, edema, and neutrophil infiltration, in the skin of TPA-treated animals.

Although further investigations are needed to clarify the exact mechanisms of these effects, our study provides compelling evidence supporting the potential of EVEs as a novel treatment for skin inflammation.

## Data Availability

All data are contained within the article.

## Supplementary Materials

The following supporting information can be downloaded at: https://www.mdpi.com/article/10.3390/ijms252312522/s1.
