# Supplementary material for: Anti-Inflammatory Effects of Extracellular Vesicles from Ecklonia cava on 12-O-Tetradecanoylphorbol-13-Acetate-Induced Skin Inflammation in Mice
Source: Int J Mol Sci. 2024 Nov 21;25(23):12522. doi: 10.3390/ijms252312522 (PMC11641720; doi:10.3390/ijms252312522)
Supplement: Supplementary file 1 [file ijms-25-12522-s001.zip › ijms-3220008-supplementary.pdf]

International Journal of
Molecular Sciences

Article

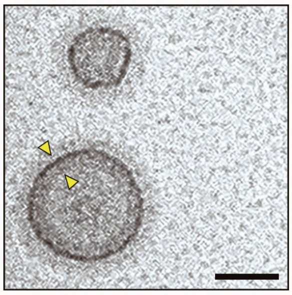

Figure S1. Cryo-transmission electron microscope images of EVEs. The yellow mark indicates a double-layered membrane. Scale bar = 200 nm. EVE, extracellular vesicle from *E. Cava*.

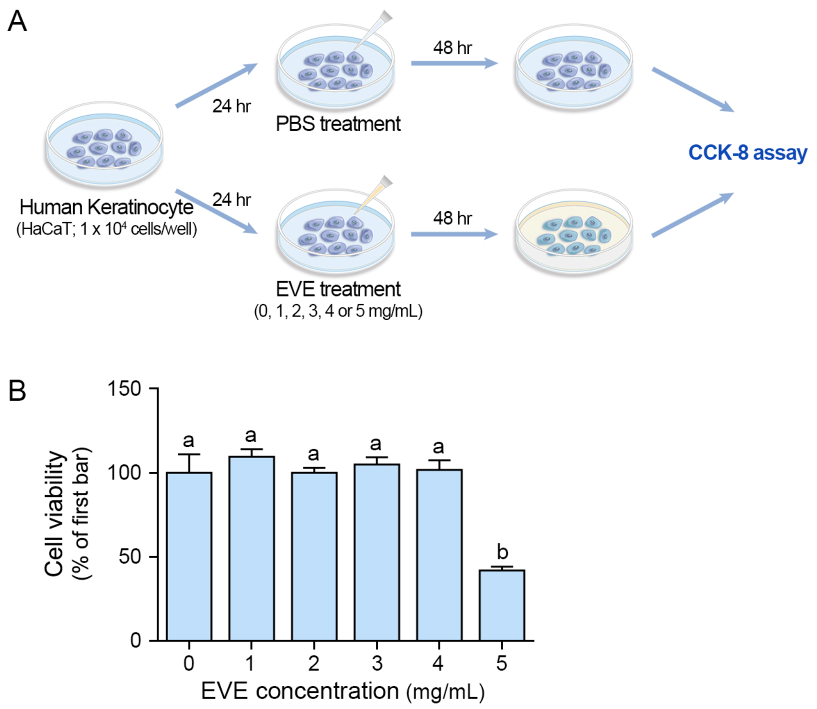

Figure S2. Cytotoxicity evaluation of EVEs in human keratinocytes. (A) Schematic diagram of EVE treatment in human keratinocytes. Human keratinocytes were treated with PBS or EVEs at concentrations of 0–5 mg/mL. (B) Cell viability after EVE treatment in human keratinocytes. Data are presented as the mean ± SD of three independent experiments. $p < 0.05$; a,b; same letters indicate non-significant differences between groups, as determined by multiple comparisons (Mann–Whitney U test). EVE, extracellular vesicles from E. cava; PBS, phosphate-buffered saline; TPA, 12-O-tetradecanoylphorbol-13-acetate.

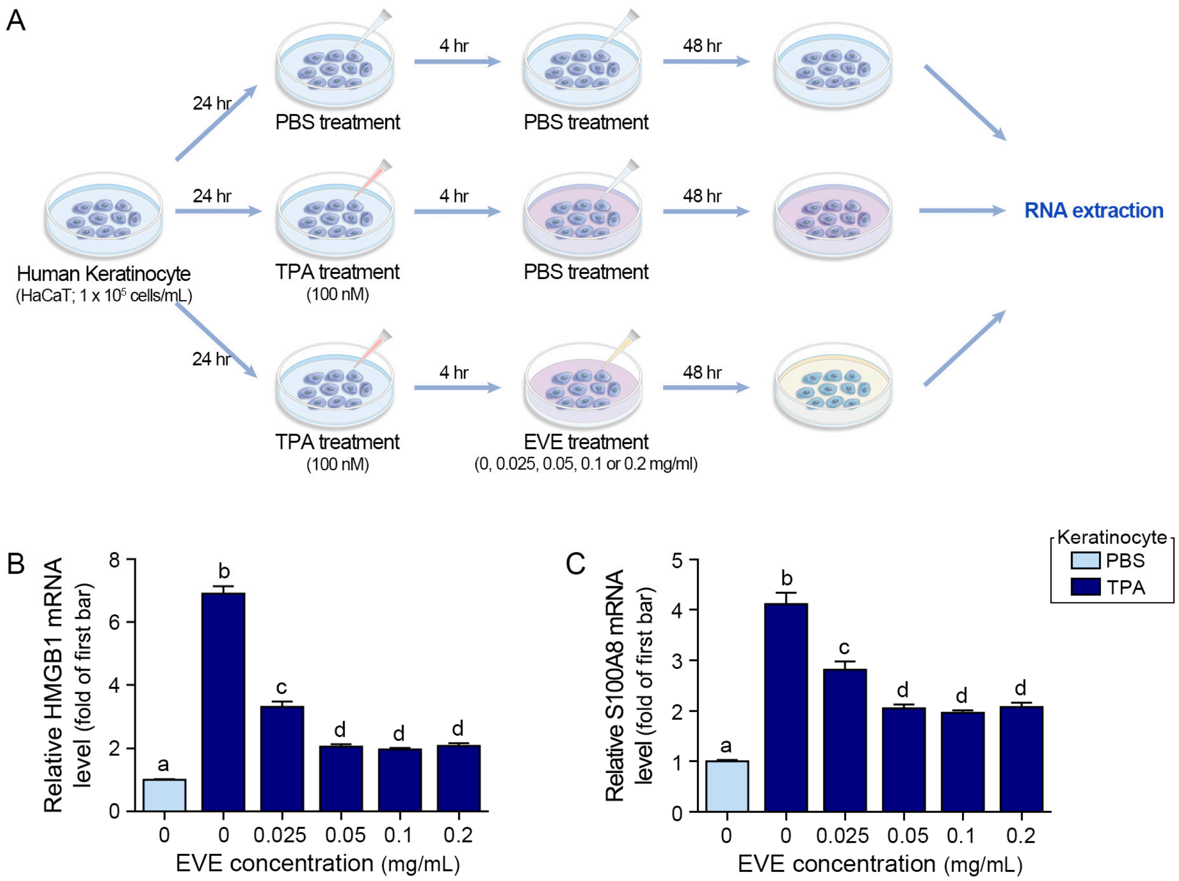

Figure S3. Evaluation of EVE treatment concentrations. (A) Schematic diagram of EVE treatment in TPA-treated human keratinocytes. Human keratinocytes were treated with 100 nM TPA for 4 h, followed by a 48 h incubation with PBS or EVEs at concentrations of 0.025, 0.05, 0.1, or 0.2 mg/mL. (B,C) Real-time quantitative PCR detection of HMGB1 (B) and S100A8 (C) mRNA expressions in TPA-treated human keratinocytes with various EVE treatment concentrations. Data are presented as the mean ± SD of three independent experiments. $p < 0.05$; a–d; same letters indicate nonsignificant differences between groups, as determined by multiple comparisons (Mann–Whitney U test). EVE, extracellular vesicles from E. cava; HMGB1, high- mobility group box-1 protein; PBS, phosphate-buffered saline; SD, standard deviation; TLR4, toll-like receptor 4; TPA, 12-O-tetradecanoylphorbol-13-acetate.

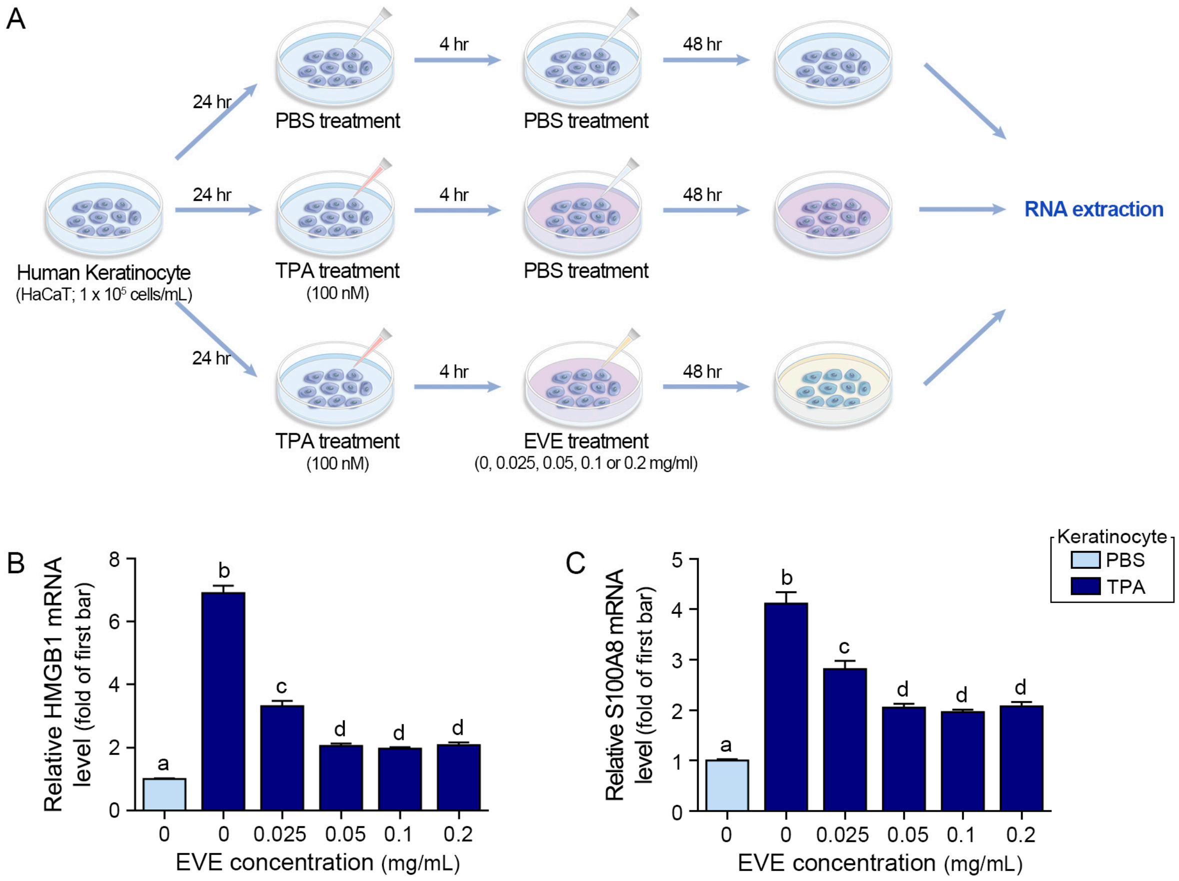

Figure S4. Schematic diagram of EVE or DXA treatment after TLR4 silencing in TPA-treated human keratinocytes. Human keratinocytes were treated with 100 nM TPA for 4 h, followed by a 48 h incubation with PBS, EVEs (0.05 mg/mL), or DXA (0.001 mM). For TLR4 knockdown, TLR4 shRNA plasmid (500 ng) was transfected for 24 h, followed by treatment with 100 nM TPA for 4 h and incubation with PBS, EVEs (0.05 mg/mL), or DXA (0.001 mM) for 48 h. DXA, dexamethasone; EVE, extracellular vesicles from E. cava; PBS, phosphate-buffered saline; TPA, 12-O-tetradecanoylphorbol-13-acetate.

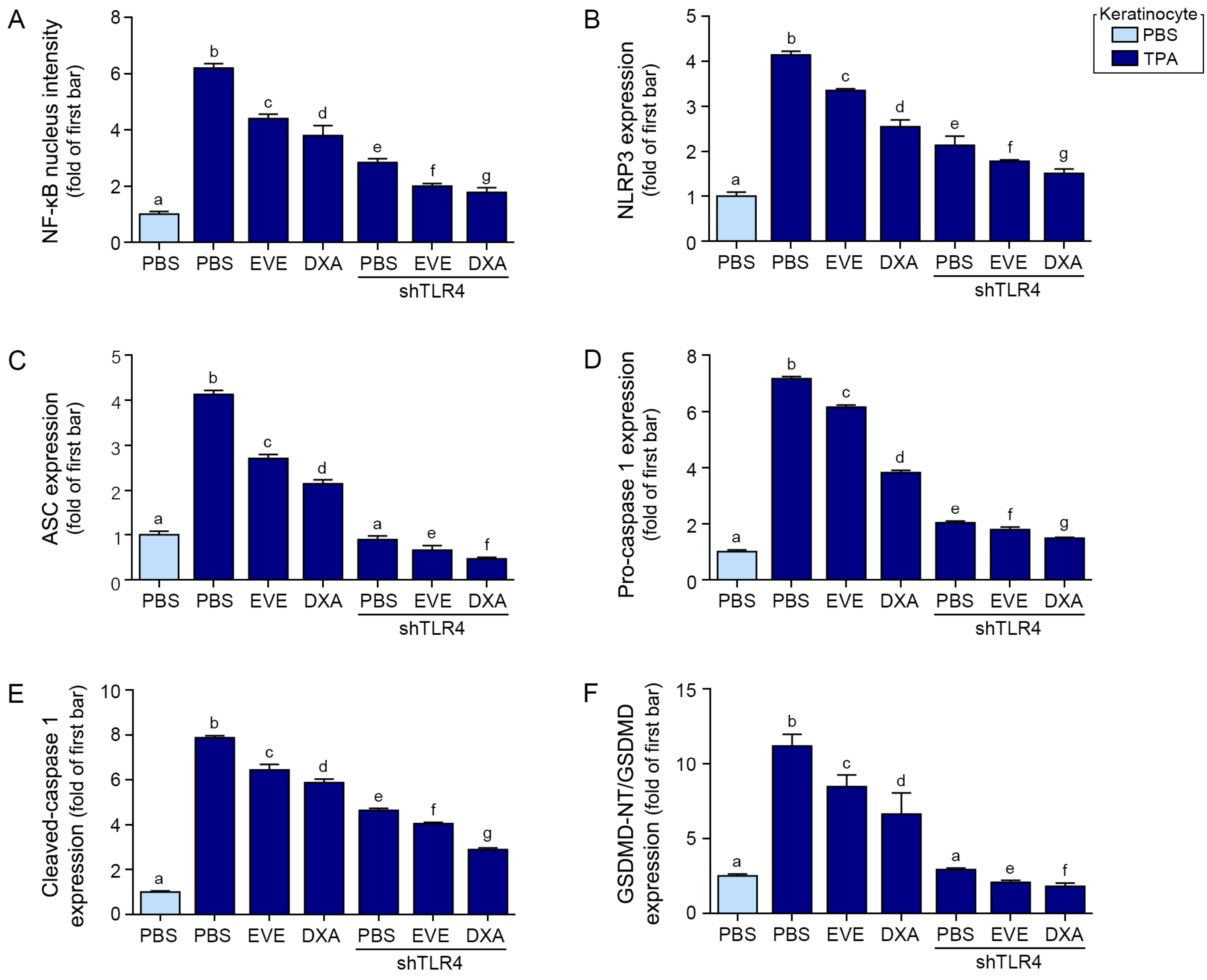

Figure S5. Quantitative evaluation of NF-κB, NLRP3 inflammasome, and pyroptosis by EVE treatment in TPA-treated human keratinocytes. (A) Quantification of NF-κB nucleus-positive cells in Figure 2A. (B–E) Quantification analysis of NLRP3 (B), ASC (C), pro-caspase 1 (D), and cleaved-caspase 1 (E) with Figure 2B using Image J software. (F) Quantification analysis of GSDMM-NT with Figure 2C using Image J software. $p < 0.05$; a–g; same letters indicate nonsignificant differences between groups, as determined by multiple comparisons (Mann–Whitney U test). ASC, apoptosis-associated speck-like protein; DXA, dexamethasone; EVE, extracellular vesicles from E. cava; GSDMD, gasdermin D; GSDMD-NT, gasdermin D N-terminal do-main; NF-κB, nuclear factor-κB; NLRP3, NOD-like receptor protein 3; PBS, phosphate-buffered saline; SD, standard deviation; TPA, 12-O-tetradecanoylphorbol-13-acetate.

Table S1. List of primers for quantitative polymerase chain reaction.

Gene *(Organism)*		Primer sequences
ACTB (*human*)	Forward	5'-GGG ACC TGA CTG ACT ACC TCA T-3′
	Reverse	5'-CCT TAA TGT CAC GCA CGA TTT-3′
HMGB1 (*human*)	Forward	5'-GGC CTT CTT CCT CTT CTG CT-3′
	Reverse	5'-GCA ACA TCA CCA ATG GAC AG-3′
S100A8 (*human*)	Forward	5'-ATA TCA GGA AAA AGG GTG CAG-3′
	Reverse	5'-CAG AAT GAG GAA CTC CTG GAA G-3′

Table S2. List of antibodies for western blot (WB), enzyme-linked immunosorbent assay (ELISA), immunocytochemistry (ICC), and immunohistochemistry (IHC).

Antibody	Company	Dilution rate		
		WB	ICC/IHC	ELISA
β-actin	Cell Signaling	1:1000	-	-
HMGB1	Abcam	1:1000	-	-
S100A8	Biorbyt	1:1000	-	-
TLR4	Novusbio	1:1000	-	-
NLRP3	Abcam	1:500	-	-
ASC	Santa Cruz	1:500	-	-
Caspase 1	Santa Cruz	1:500	-	-
GSDMD	Santa Cruz	1:1000	-	-
GSDMD-NT	Cell Signaling	1:1000	-	-
NF-κB	Cell Signaling		1:100	-
IL-18	Invitrogen	-	-	1:500
IL-1β	Bioss	-	-	1:500
